# Mobile applications to prescribe physical exercise in frail older adults: review of the available tools in app stores

**DOI:** 10.1093/ageing/afad227

**Published:** 2023-12-28

**Authors:** Luis Soto-Bagaria, Sophie Eis, Laura Mónica Pérez, Lorena Villa-García, Oriol de Solà-Morales, Carme Carrion, Maria Giné-Garriga, Marco Inzitari

**Affiliations:** REFiT Aging Research Group, Parc Sanitari Pere Virgili and Vall d’Hebron Institute of Research (VHIR), Barcelona, Spain; SAFE Research Group, Faculty of Psychology, Education and Sport Sciences Blanquerna, Ramon Llull University, Barcelona, Spain; Fundació HiTT, Barcelona, Spain; REFiT Aging Research Group, Parc Sanitari Pere Virgili and Vall d’Hebron Institute of Research (VHIR), Barcelona, Spain; REFiT Aging Research Group, Parc Sanitari Pere Virgili and Vall d’Hebron Institute of Research (VHIR), Barcelona, Spain; Department of Medicine, Universitat Autònoma de Barcelona (UAB), Barcelona, Spain; Qida, Sabadell, Spain; Department of Public Health, Mental Health and Mother-Infant Nursing, Faculty of Nursing, University of Barcelona, L'Hospitalet de Llobregat, Barcelona, Spain; Fundació HiTT, Barcelona, Spain; Ehealth Lab Research Group, School of Health Sciences, Universitat Oberta de Catalunya, Barcelona, Spain; SAFE Research Group, Faculty of Psychology, Education and Sport Sciences Blanquerna, Ramon Llull University, Barcelona, Spain; Department of Physical Activity and Sport Sciences, Faculty of Psychology, Education and Sport Sciences (FPCEE) Blanquerna, Ramon Llull University, Císter 34, Barcelona 08022, Spain; REFiT Aging Research Group, Parc Sanitari Pere Virgili and Vall d’Hebron Institute of Research (VHIR), Barcelona, Spain; Ehealth Lab Research Group, School of Health Sciences, Universitat Oberta de Catalunya, Barcelona, Spain

**Keywords:** applications, frail, older, physical exercise, healthy ageing, systematic review, older people

## Abstract

**Introduction:**

Different remote interventions, such as applications (apps), have been used to continue promoting healthy ageing and preventing disability during the COVID-19 pandemic. The growing trend of apps in health is exponential and may facilitate scaling up physical activity prescription. Numerous tools are available, but little is known regarding their appropriateness, validation and recommendation, especially for frail older adults.

**Methods:**

In-house, we developed an application that makes both the Apple app Store and the Google Play Store searchable using topic-related keywords and facilitates the extraction of basic app-information of the search results. The study was aimed at apps available to an English-speaking market. The resulting apps were filtered using various inclusion and exclusion criteria. The resultant apps underwent a more in-depth characterisation and searches for scientific publications on each app website and PubMed.

**Results:**

From an initial search result of >2,800 apps, 459 met the initial inclusion criteria. After a more in-depth review of their features, 39 apps remained for possible app in older frail patients. After testing them, 22 apps were excluded. Seventeen apps fit the inclusion and exclusion criteria and were deemed appropriate after peer review. Of these, only one app, Vivifrail, had any type of publication/published evidence.

**Conclusion:**

Apps can be valuable tool in prescribing exercise for frail older adults living in the community. However, few apps seem useful on a large scale, and there is limited evidence to support their effectiveness. It is important to invest in adapting Information and Communication Technologies to this population group.

## Key Points

- Digital solutions through apps aimed at prescribing physical exercise in frail older adults are poorly adapted.
- Only one app (Vivifrail) met the needs of frail older adults and showed to be evidence-based.
- There is the need to invest in a specific, more tailored and accessible app targeting this population group.
- For empowered users, the majority of Apps are dynamic and can adapt to the user's needs at any given time, can conduct self-assessments and even promote intergenerational usage.
- Digital exclusion is a reality nowadays for older adults; policies to reduce it, which should adopt co-design strategies, are urgent.

## Background

The population is ageing worldwide, and new challenges need to be overcome to improve older adults’ health and reduce the global burden of communicable diseases [1]. Regular physical activity (PA) prevents non-communicable diseases, such as cardiovascular diseases, cancer, diabetes and mental health disorders, and improves their management [2–4], which suggests that increasing PA is one of the most promising strategies. Nevertheless, according to the World Health Organization (WHO), one in four adults do not meet the recommended levels of PA; their risk of death by noncommunicable diseases is 20–30% higher in comparison with sufficiently active people [4].

In people over 65 years of age, the WHO defined sufficient levels of PA as 150 minutes of moderate PA or 75 minutes of vigorous PA per week, or a combination of the two. The benefits are further increased if weekly PA is doubled [5]. For additional health benefits, it is important to note that older people should do muscle-strengthening activities involving all major muscle groups at least twice a week, and functional balance and flexibility exercises at least 3 days a week. Physical exercise (PE) is planned, structured and repetitive PA that aims to improve physical fitness [6]. Solid and robust scientific evidence from experimental [7–10] and real implementation programmes [11] suggests that PE is key to improving intrinsic capacity (i.e. the combination of all physical and mental capabilities that an individual can use at a given time [12]). Supporting intrinsic capacity is essential to reversing frailty and postponing disability and dependency.

The COVID-19 pandemic and subsequent lockdown measures have decreased PA levels in the population [13–16] with negative consequences on health, particularly in older adults. Since March 2020, many healthcare and social services have increased their electronic health (eHealth) interventions (also referred to as ‘digital health interventions’) to promote healthy ageing and prevent disability during and after the COVID-19 pandemic [17]. Evidence suggests that mobile health (mHealth), as a form of eHealth intervention, contributes to promoting PA [18] in older adults and may facilitate the implementation, evaluation and scaling of PE prescriptions and PA-based interventions.

Recent years have seen growth in the development of mobile apps that seek to increase PA levels and improve health outcomes. Many eHealth apps, with varying features, quality and security standards, are available in app stores. Older adults, caregivers and healthcare professionals could use or prescribe the use of these apps. However, there is a scarcity of evidence-based assessments of their usability and validity, especially focused on frail older adults. Thus, usage and adherence to these apps by the adult population, as well as by the professionals able to prescribe them, may be limited [19].

These aspects are particularly relevant considering that the digital divide is exacerbated with age and is particularly pronounced in older adults with lower education, lower income or worse health condition [20, 21]. A recent study assessing older adults’ readiness to adapt to and use telemedicine during the COVID-19 pandemic found that 38% of surveyed older adults were not ready for video visits, predominantly due to inexperience with technology [20].

We aimed to review and evaluate whether the apps whose main objective was to improve performance of PE and enhance movement, and which are available in the most popular app stores, were specifically designed for older adults and met the needs of the frail older adults (in terms of content and usability). A secondary aim was to assess the evidence that supports them.

## Methods

We conducted a systematic search for smartphone apps in accordance with the Preferred Reporting Items for Systematic Reviews and Meta-Analyses (PRISMA) [22]. Although it is not entirely clear if these guidelines may be a valid framework for reviews focused on eHealth solutions and apps (Grainger et al., 2020) [19], there is no gold standard for the systematic search and evaluation of apps. The first step of the review was to search for the apps in the app stores. Subsequently, a literature review was conducted to find scientific evidence supporting the use of them.

### Search strategy

To explore the landscape of mobile apps available for PE prescription in older adults, a systematic search was conducted in the Apple app Store and Google Play platforms using the following terms: ‘older adult’, ‘therapeutic exercise’, ‘ageing’, ‘frailty’, ‘elderly’, ‘fall risk’, ‘physical activity’ and ‘balance’.

To facilitate structured data extraction from the app stores and enable easy filtering and sorting of search results, an in-house *ad hoc* search tool was developed. It makes the stores searchable and produces spreadsheets containing the following information: app name; app developer; approximate number of downloads (available only in Google Play) and number of user ratings (available only in Apple app Store); average user rating; category; price; release date; corresponding URL and language (available only in Apple app Store). Our tool is not commercially available, has been only used for this research, and has been limited to extracting up to 200 of the most relevant apps for each search. The search engine allows the querying of key terms in a particular country’s app store, therefore granting access to potentially region-specific apps. This study restricted its search to the following English-speaking countries: Australia, Canada, Ireland, South Africa, United Kingdom and the United States. The search terms were used in English between the dates of 01 June 2021 and 15 June 2021.

### Eligibility criteria

We conducted this section as shown in a previous article [23]. After generating spreadsheets of results for each search term, results were compiled by country into master files. We kept only apps which had >10,000 downloads to avoid including apps that had been used for specific research studies with no further use in clinical/professional practice and were classified in the ‘Health & Fitness’and ‘Medical’categories. For apps available in Apple app Store, no restriction was applied contingent on number of downloads, since this information was not provided and rating amounts did not provide a satisfactory proxy of downloads. Then, country-specific results were compiled into one overall master file. The apps had to be aimed at older people.

### Exclusion criteria

#### Exclusion criteria

We manually removed all duplicate apps and those that hadn’t been updated within the last 3 years to exclude those that were no longer actively managed and maintained, applications need updates or modifications over time to adapt to new operating systems and fix possible bugs reported by users.

To further refine the results, we defined various exclusion terms (listed in Annex 1). Including these terms in the search would generate a volume of apps that are not relevant to the objective of the review. In our case, we were not interested in focusing on anxiety or depression and related topics, such as meditation. Other notable terms that have been excluded are those related to nutrition and weight loss, sleep or specific exercises like Yoga. For obvious reasons, anything related to children and sports (soccer, hockey, etc.) was excluded.

We then excluded the apps that were explicitly unavailable in English or were not targeted at older adults.

Finally, we exclude the apps were not supported by science (that there are no publications that support the use of the app in our target population).

#### Selection process

Two researchers, L.S. and S.E., with previous experience in digital health and PE prescription for older adults, carried out the app selection process in three phases.

In the first phase, each researcher independently evaluated the retrieved app’s names according to the eligibility criteria. The resulting apps underwent three rounds of peer review to filter out those that did not fit the topic, based on their names. In the second phase, a review of the remaining apps’ websites was conducted to ascertain relevance, and unfitting ones were sorted out by L.S. and S.E. separately. In the third phase, L.S. and S.E. performed a final check of the apps’ websites, and the selected apps were downloaded for a complete evaluation of their features and eligibility. A standardised form designed for this study was used to record the features of the selected apps. Their results were compiled, compared and discussed as necessary to understand any overlaps and discrepancies. If disagreements appeared during the selection process, the two researchers conducted an exhaustive review until a mutual agreement was reached.

### Analysis

The filtered apps were then systematically analysed in more detail to understand the specific app type, components, target audience and objectives. Within each app, we reviewed the following features, as detailed hereafter: education, self-monitoring, goal-setting, feedback, gamification, adaptation and progression, and availability of a peer network [23]. Each element was then marked as either present or absent (SE, LS). ‘Education’ refers to any informative elements that educate the user on the correct form and practice of exercises, frailty or healthy ageing in general. ‘Self-monitoring’ refers to the in-app possibility of recording and assessing performance and progress throughout a PE regimen. ‘Goal setting’ was defined as the in-app option to set personal specific goals that should be achieved in a given timeframe. ‘Feedback’ refers to any interactive elements that allow the user to receive feedback in-app regarding their performance, progress, form, etc., potentially coupled with advice on how to continue the regimen. The dimension of ‘gamification’ checked whether the exercises had been made more interactive or attractive by, for example, attaching scores to completed exercises and encouraging daily use through game-like mechanisms. ‘Adaptation and progression’ builds on the concept of feedback, meaning that the app is able to adjust the PE or treatment plan according to the needs and performance of the user. Lastly, the ‘peer network’ dimension refers to the possibility of exchanging or sharing messages, achievements, etc. with other app users.

### Scientific validation of the apps

A targeted literature search was performed to detect whether any of the selected apps can support their acceptability, validation, use, effectiveness, etc. with scientific evidence. We conducted a literature search in the databases MEDLINE (Pubmed) and ClinicalTrials.gov, from their inception until October 2021. For the search in MEDLINE (PubMed), we input each app’s name in combination with the following keywords: ‘(App) OR (application) OR (mobile)’. No further filters or search criteria were applied to the PubMed search.

The reference sections of the eligible papers were manually reviewed to identify additional studies. Furthermore, each app’s developer’s website was examined, looking for references to be included.

## Results

The mobile app search yielded 11,719 results: 3320 apps in the Apple app Store and 8,399 in Google Play. Once duplicates were removed, a total of 2,864 apps were found to be potentially eligible. After the title review, 2,405 were excluded based on the pre-set criteria. Finally, 15 apps were included in this review. Only one of the selected apps, Vivifrail, had a research base of its clinical use in frail and/or older people [24–26].

**Figure 1:**
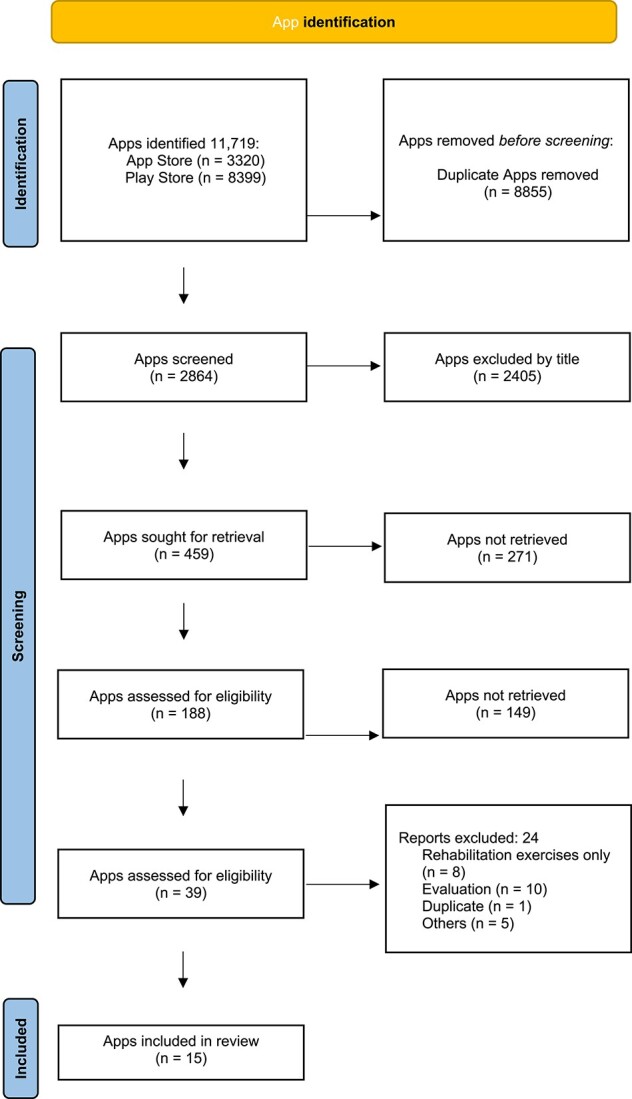
Flow-chart of the applications included in this review.

Figure 1 shows the flowchart of app results obtained at various stages of filtering and applying inclusion and exclusion criteria.

Table 1 displays the features of the 15 apps included in this review.

**Table 1:** Summary of the characteristics of the selected applications

**App**	**Developer**	**Components**	**Objective**	**User/Target**	**Elements included**						
					Education	Self-monitoring	Goal setting	Feedback	Gamification	Adaptation/ Progression	Peer network
A45Plus Pro-Ageing Empowerment	Didier Quintard	Application only (pay to access all features)	Exercises and nutritional advice	Women over the age of 45	✓	✓	✗	✗	✗	✓	✗
Ageing Well Fitness	WaterART Fitness International Inc	Application only	(Water) exercise and self-care videos	Older adults	✓	✓ (steps only)	✓	✗	✗	✗	✓
Daily Senior Fitness Exercise	EBMACS	Application only	Provides exercise instructions with photos according to body part	Older adults	✗ (only exercise explanation)	✗	✗	✗	✗	✗	✗
Workout for Over 50’	Fitric	Application only (pay to unlock all features)	Provide simple exercises and plans lasting 1–2 months	Older adults	✗ (only exercise explanation)	✓	✗	✗	✗	✓	✗
Exercise plan for seniors	Stay Fit With Samantha	Application only (pay to unlock all features)	Provide simple exercises and plans lasting 1–2 months	Older adults	✗ (only exercise explanation)	✓	✗	✗	✗	✓	✗
Healure: Physiotherapy Exercise Plans	Healure Technology	Application only	Provides exercise plans with video instruction according to ailment	Anybody	✗ (only exercise explanation)	✓	✗	✗	✗	✓	✗
MedBridge GO for Patients	MedBridge	Application only	Provides exercise plans with video instruction	Anybody working together with physiotherapist	✓	✓	✓	✓	✓	✓	✗
Moves4Me	Digital Health and Well-Being Limited	Application and website	Exercises (videos) and tracking/assessment	Older adults	✗	✓	✗	✗	✗	✓	✗ (forum associated with app)
Physiotec	Physiotec	Application to coordinate with physio-therapist	Rehabilitation exercise software incl. Online exercise prescription service	Anybody working together with physiotherapist	✗	✓	✓	✓	✗	✓	✗
Physiotherapy Exercises	adminapps	Application only	Provides exercise instructions with photos	Anybody	✗ (only exercise explanation)	✗	✗	✗	✗	✗	✗
Senior Fitness - Home workout for old and elderly.	K2 Labs	Application only	Provides exercise videos	Older adults	✗ (only exercise explanation)	✓	✗	✗	✗	✗	✗
Seniors Beginner Workout - 20 Minutes Training	App4Life dev	Application only	Provides exercise instructions with photos/gifs	Older adults	✓	✗	✗	✗	✗	✗	✗
SitFit Exercise	Candlhat Studios	Application only	Seated exercises	Older adults	✗ (only exercise explanation)	✗	✗	✗	✗	✗	✗
TherEx Anywhere	Skyscape Medpresso Inc	Application (pay for full access)	Physical therapy exercises and exercise plan creation	Anybody	✗ (only exercise explanation)	✗	✓	✗	✗	✓	✗
Vivifrail	Vivifrail	Application only	Classification, exercise provision, monitoring	Patients and/or patient managers	✓	✓	✓	✗	✗	✓	✗

None of the selected apps included all the assessed characteristics. MedBridge GO included the most elements, with 6 out of 7, only missing the peer network. In fact, only the Ageing Well Fitness app integrated this feature. Furthermore, MedBridge GO was the only app that included feedback and gamification features, the rest had a very limited level of interactivity. Of the apps that included some type of informative element, only eight included instructions on how to perform the given exercises, but did not include information regarding healthy ageing, frailty, information specific to older adults, etc. Various apps, such as Daily Senior Fitness Exercises or Senior Fitness—Home Workout, offered very limited services, mainly only sets of exercises with some instruction on how to do them, without any goal setting, progress tracking or feedback functionalities.

Only one of the selected apps, Vivifrail, had a research base of its clinical use in frail and/or older people [24–26]. Analysing the methodological quality of the studies, we highlighted three RCTs (one of them multicentre) with significant samples and positive results in the application of their protocol. Two independent investigators assessed the quality of the app through the Mobile Application Rating Scale [27] scale. This scale, which hast excellent inter-rater reliability and internal consistency [27], includes 23 items that assess engagement, functionality, aesthetics, information quality and subjective quality. The items were rated 1 (inadequate) to 5 (excellent). The Vivifrail app was given a score of 3.98 in quality and 4.25 in subjective quality. The best-rated section was functionality (4.5), followed by information (4.25), subjective app quality (4.25), engagement (3.7) and aesthetics (3.5).

The Physiotec app does not yet have any associated publications; however, two ongoing trials involving the app were found on ClinicalTrials.gov (identifiers: NCT05140226, NCT04945356), both first registered in 2021. The first one (NCT05140226) discusses cognitive and physical training in patients with chronic obstructive pulmonary disease and the second one (NCT0494535) discusses telerehabilitation during pandemic containment measures of COVID-19. Neither of them focusses on our population group.

## Discussion

After a systematic search and review, only 15 apps met the inclusion/exclusion criteria. Only Vivifrail was found to be evidence-based and meet the needs for both content (no prescription required from a professional, adaptable, free, with the possibility of progression, and providing both written and audiovisual information) and usability of the frail older population. Six were paid; of these, five required payment to access all the app features (videos, exercises, routines, etc.) and one requested the payment to be made to the professional who would design the training routine exercises in-app. Three apps were based on classic rehabilitation of functional deficits, not in PE, and required a prior prescription of exercises by a therapist.

To our knowledge, this is the first study assessing PE apps for frail older adults. McGarrigle *et al.* (2020) reviewed the digital approaches to driving engagement of older adults in strength and balance exercises. This study recommended high-quality apps and websites, evaluated in a methodological, exhaustive and detailed way, thus being potentially able to replace face-to-face interventions. However, the authors’ research was focused on the United Kingdom, so their results cannot be generalised. Even though there are no quality studies in the literature to support app use in this frail population, they seem to facilitate doing more exercises at home [28]. In our analysis, we include three apps that were excluded in McGarrigle *et al.*’s search due to the lack of scientific evidence (Exercise Plan for Seniors, Moves4me and Senior Beginner Workout). Reyes *et al.* (2018) reviewed apps that promote balance for older adults, and only found apps for iOS (App Store). All of them showed good quality, emphasising that older adults may not always need to attend in-person rehabilitation services [29]. There was no overlap with the apps in this review. The authors pointed out that the overall patient confidence towards virtually delivered healthcare services was low, but as this study was published before the COVID-19 pandemic, these feelings may have changed and evolved towards a better outcome.

In line with Klimova’s (2018) results, we found that despite many applications targeting older adults, they do not seem adapted to their physical and/or cognitive needs [30], so they would not have been specifically designed to target their needs. The simple fact of requiring an email account for registration is currently a great limitation for this population group, as well as requiring an internet connection or a smartphone.

We have not found apps that adapt the exercise routines to the cognitive function of the older adults. As an example, apps may need to include a screening assessment that enables providing more targeted exercises. In terms of exercise prescription, the components of intensity, frequency, volume and progression, which are key in the implementation of an exercise programme, were not present in any of the apps reviewed. To achieve an optimal stimulus when exercise is prescribed, it is important to ensure that the intensity (weight used, number of series and repetitions, rest time, progression), the volume (total amount of work performed for a period of time) and the frequency (amount of sessions a week) are personalised and adapted to each person and their physical situation. In addition, if a progression of these variables is not guaranteed, an optimal stimulus may cease to be optimal due to adaptation of the musculoskeletal system.

An app for frail older adults should be simple, intuitive and focused on their needs, e.g. in case of visual impairment, a touchscreen may present a challenge, and icons and font sizes should be bigger [31]. Given the heterogeneous socio-economic status of older adults, mechanisms to reduce inequalities should be considered. On the simple and intuitive side, it would be interesting to include end-users in the development and testing phase via a co-creation process. Frail older people from different backgrounds and with different technological knowledge should be involved.

When caring for frail older adults, other risk factors and interventions besides exercise should be taken into account [32] and complement the app by adding some type of interventions to improve nutrition, PA levels, socialisation, sedentary lifestyles, etc [32]. Gamification can help to improve socialisation, an extremely important aspect in the ageing process [33], improving social connections [34]; moreover, it might increase adherence to PE [35].

Another important aspect is professional feedback, in particular for older adults: providing training and support is crucial when prescribing an app [31]. Offering the possibility of opening a line of communication creates a safe environment, in addition to being able to follow up, send reinforcement messages and modify the recommendations as needed without a physical visit.

The applicability of using apps for frail older adults lies in having an additional tool in prescribing exercise in our daily clinical practice. Apps, for the most part, being dynamic, can adapt to the user’s needs at any given time, conduct self-assessments and even promote intergenerational usage. Furthermore, we can reach individuals who have difficulty leaving their homes.

We are aware that digital exclusion is a reality nowadays [36]. The majority of apps are designed with minimal knowledge assumed and do not cater to the needs of users without experience, knowledge, and with certain comprehension difficulties in this field. While it is true that we are encountering more older adults using technology every day [37], especially after the pandemic, we are still far from being able to reach the majority of the population. Even apps for this age group seem to be designed for someone else to manage, distancing them from empowerment and self-responsibility in the process of healthy ageing.

We must acknowledge the different limitations of our study. First of all, the development and market speeds of technology are overall very fast; therefore, we cannot discount the possibility that new applications were launched on the market during the publication of our manuscript. Second, our search was conducted in the Google Play and App Store platforms, which left aside other possibilities such as web pages. However, we searched for apps on both Google Play and app Store in different countries using an *ad hoc* algorithm. Third, there is a lack of methodology to be followed for this kind of reviews; we have adapted our strategy from a classic systematic review (PRISMA), but not all the steps are applicable to our type of research.

## Conclusion

From our search, only one app (Vivifrail) meets the needs of the older people with frailty and is evidence-based. However, digital solutions through apps for the prescription of PE in this context are poorly adapted to this specific, growing population. Being aware of the great importance of exercising for older adults, it would be advisable for the future to invest in a broader, more tailored and accessible app offer for this population.

## Supplementary Material

aa-23-0608-File002_afad227
