# Supplementary material for: Mobile applications to prescribe physical exercise in frail older adults: review of the available tools in app stores
Source: Age Ageing. 2023 Dec 28;52(12):afad227. doi: 10.1093/ageing/afad227 (PMC10756334; doi:10.1093/ageing/afad227)
Supplement: aa-23-0608-File002_afad227 [file aa-23-0608-file002_afad227.docx]

Annex 1. Excluded terms

| Anxiety |  | Period | 30 day | Abs | Step |
| --- | --- | --- | --- | --- | --- |
| Depression | Water | Skin | Kid | Box | Gym |
| Meditat* | Calori* | Medic* | Pet | Butt | Posture |
| Mindful | Diet | Food | Loss | Cycl* | Basketball |
| Dementia | Nutri* | Sleep | Log | HIIT | Football |
| Mental | Breath | Tracker | Fat | Run | Hockey |
| Mind | Reminder | Time | Splits | Yoga | Soccer |
